# Publisher Correction: PRKCSH contributes to tumorigenesis by selective boosting of IRE1 signaling pathway

**DOI:** 10.1038/s41467-019-11824-3

**Published:** 2019-08-16

**Authors:** Gu-Choul Shin, Sung Ung Moon, Hong Seok Kang, Hyo-Sun Choi, Hee Dong Han, Kyun-Hwan Kim

**Affiliations:** 1 Department of Pharmacology, Center for Cancer Research and Diagnostic Medicine, IBST, School of Medicine, Konkuk University, 1 Hwayang-Dong, Kwangjin-Gu, Seoul, 143-701 Republic of Korea; 2 Center for Advanced Bioinformatics & Systems Medicine, Sookmyung Women’s University, Hyochangwon-Gil 52, Youngsan-Gu, Seoul, 140-741 Republic of Korea; 3 Department of Immunology, School of Medicine, Konkuk University, Chungwon-Daero 268, Chungju, Republic of Korea; 4 Research Institute of Medical Sciences, Konkuk University, 1 Hwayang-Dong, Kwangjin-Gu, Seoul, 143-701 Republic of Korea

**Keywords:** Tumour biomarkers, Apoptosis, Cell signalling, Stress signalling

Correction to: *Nature Communications* 10.1038/s41467-019-11019-w, published online 18 July 2019.

The original version of this Article contained errors in Figs. 3, 4 and 7. In the original version of Fig. 3, panel **e** was incorrectly labelled as panel **f**, and the panel label on panel **f** was omitted.

In the original version of Fig. 4i, the *y*-axis label of the graph second from the left incorrectly read ‘*Total XBP1* mRNA (2^−ΔΔCT^)’ rather than the correct ‘*Spliced XBP1* mRNA (2^−ΔΔCT^)’.

In the original version of Fig. 7f, the *y*-axis label of the graph incorrectly read ‘Tumour volume (mm^2^)’ rather than the correct ‘Tumour volume (mm^3^)’.

This has been corrected in both the PDF and HTML versions of the Article.

