# Supplementary material for: PRKCSH contributes to tumorigenesis by selective boosting of IRE1 signaling pathway
Source: Nat Commun. 2019 Jul 18;10:3185. doi: 10.1038/s41467-019-11019-w (PMC6639383; doi:10.1038/s41467-019-11019-w)
Supplement: Supplementary file 1 — Supplementary Information [file 41467_2019_11019_MOESM1_ESM.doc]

**Supplementary Information**

**PRKCSH Contributes to Tumorigenesis by Selective Boosting of IRE1 Signaling Pathway**

**(Shin et al.)**

**Supplementary Figures**

**
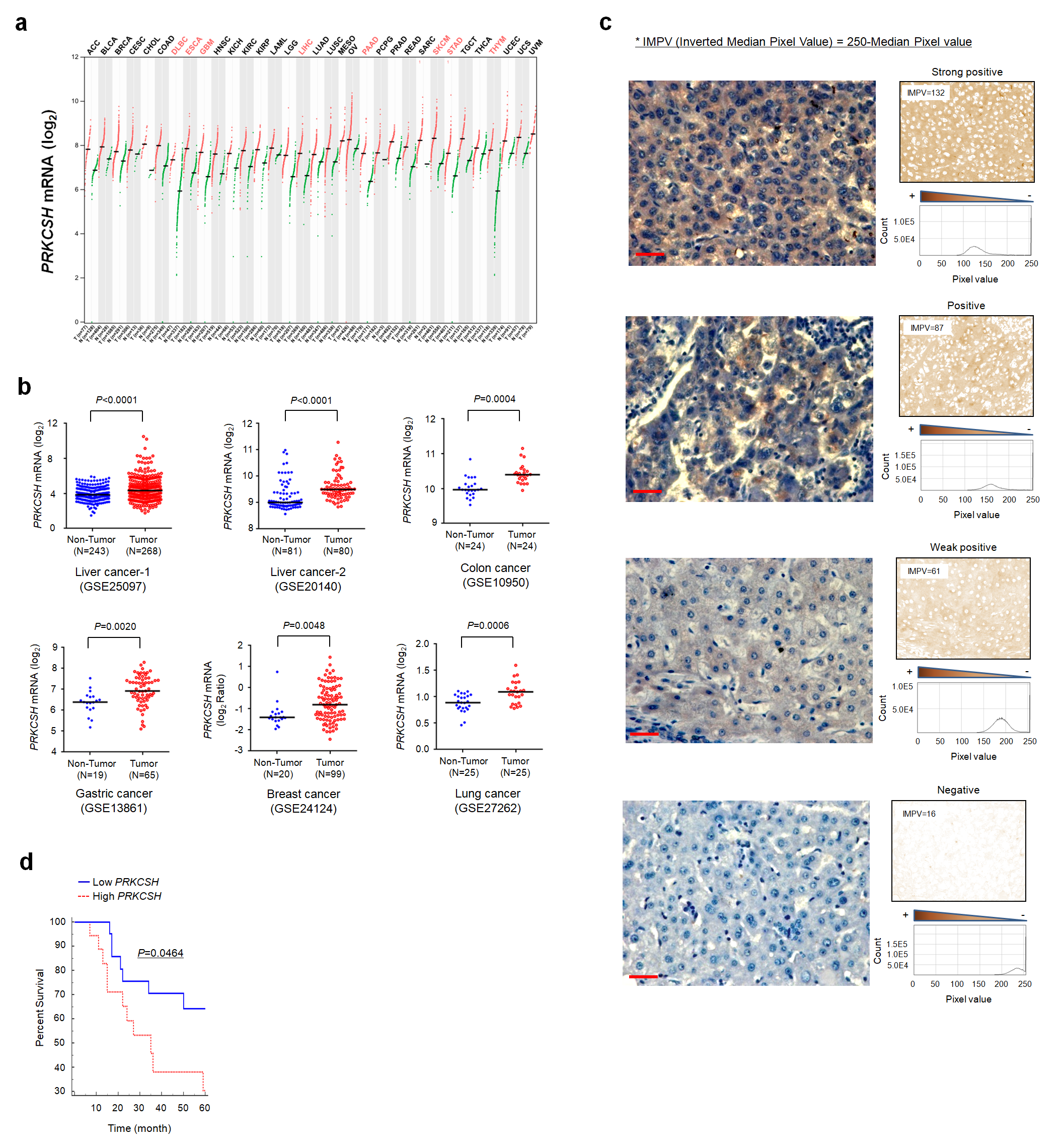
**

**Supplementary Figure 1** (related to Fig. 1). Analysis of PRKCSH expression in human tumor tissues. **a** *PRKCSH* mRNA expression was analyzed by using gene expression data sets from TCGA and the GEPIA web tool. ACC, Adrenocortical carcinoma; BLCA, bladder urothelial carcinoma; BRCA, breast invasive carcinoma; CESC, cervical squamous cell carcinoma and endocervical adenocarcinoma; CHOL, cholangio carcinoma; COAD, colon adenocarcinoma; DLBC, lymphoid neoplasm diffuse large B-cell lymphoma; ESCA, esophageal carcinoma; GBM, glioblastoma multiforme; HNSC, head and neck squamous cell carcinoma; KICH, kidney chromophobe; KIRC, kidney renal clear cell carcinoma; KIRP, kidney renal papillary cell carcinoma; LAML, acute myeloid leukemia; LGG, brain lower grade glioma; LIHC, liver hepatocellular carcinoma; LUAD, lung adenocarcinoma; LUSC, lung squamous cell carcinoma; MESO, mesothelioma; OV, ovarian serous cystadenocarcinoma; PAAD, pancreatic adenocarcinoma; PCPG, pheochromocytoma and paraganglioma; PRAD, prostate adenocarcinoma; READ, rectum adenocarcinoma; SARC, sarcoma; SKCM, skin cutaneous melanoma; STAD, stomach adenocarcinoma; TGCT, testicular germ cell tumors; THCA, thyroid carcinoma; THYM, thymoma; UCEC, uterine corpus endometrial carcinoma; UCS, uterine carcinosarcoma; UVM, uveal melanoma. **b** The levels of *PRKCSH* mRNA in human tumor tissues obtained from microarray data from the NCBI GEO database. Scatter plots show the relative levels of *PRKCSH* mRNA in non-tumor versus tumor tissues. The median expression levels in each group are indicated by horizontal lines. Significance of the differences between non-tumor and tumor tissues was determined by the Student *t*-test. **c** Immunohistochemical analysis of PRKCSH protein in liver tissues. The staining intensity of PRKCSH protein was measured by using ImageJ and IHC Profiler software as described in Methods. Scale bars represent 20 µm. **d** Kaplan–Meier survival curves for patient groups with high and low *PRKCSH* expression. The microarray data was obtained from the EMBL-EBI database (E-TABM-36). *P* value was calculated by Log-rank test.


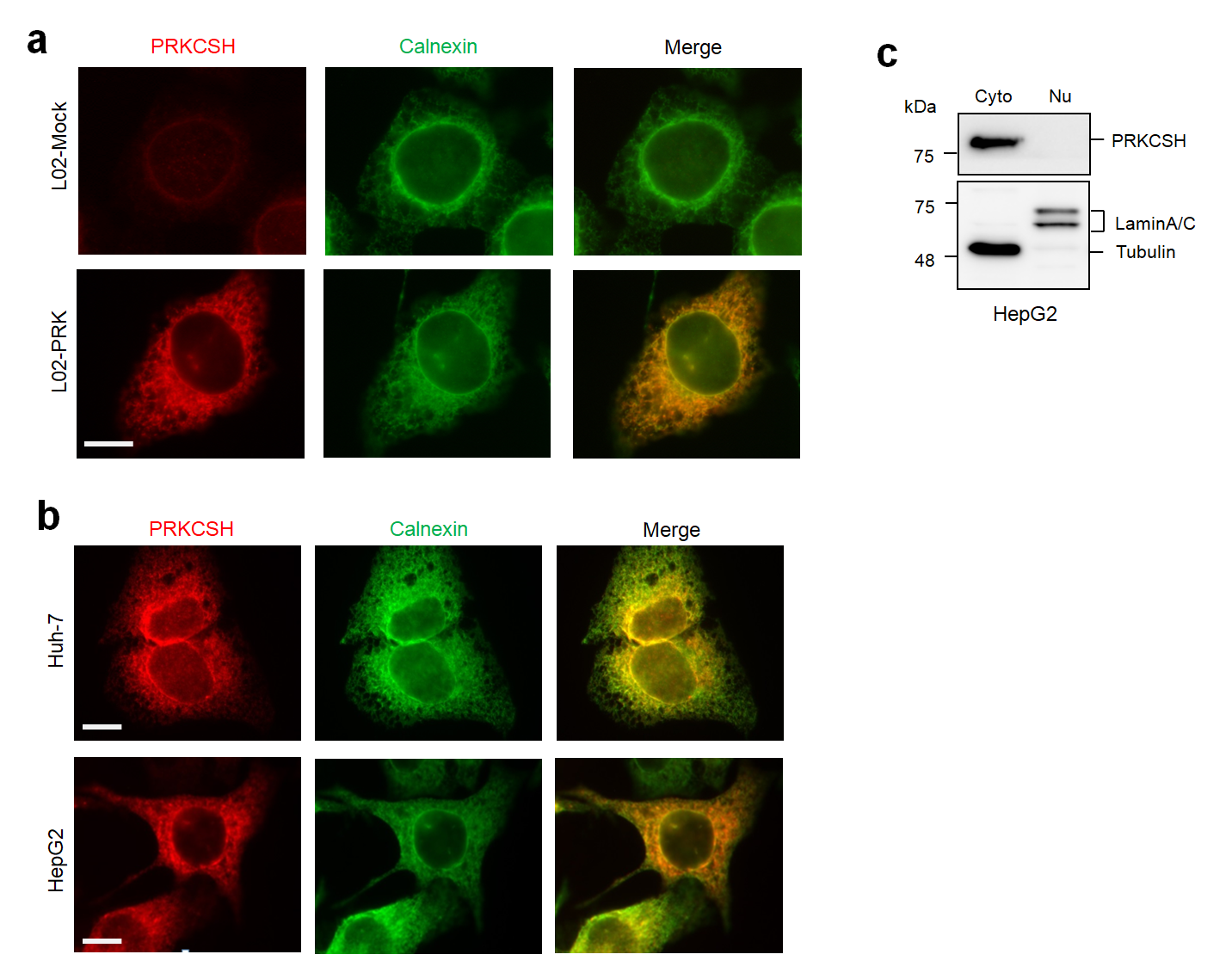


**Supplementary Figure 2** (related to Fig. 2). Intracellular localization of PRKCSH in liver cell lines. **a, b** Immunocytochemical analysis of ectopically expressed PRKCSH in L02-PRK cells (a), or HepG2 and Huh-7 cells (b). Calnexin was used as an ER marker. Scale bars represent 5 µm. **c** Analysis of subcellular fractions for the presence of PRKCSH. Lamin A/C and tubulin were used as a nuclear and cytoplasmic marker, respectively.


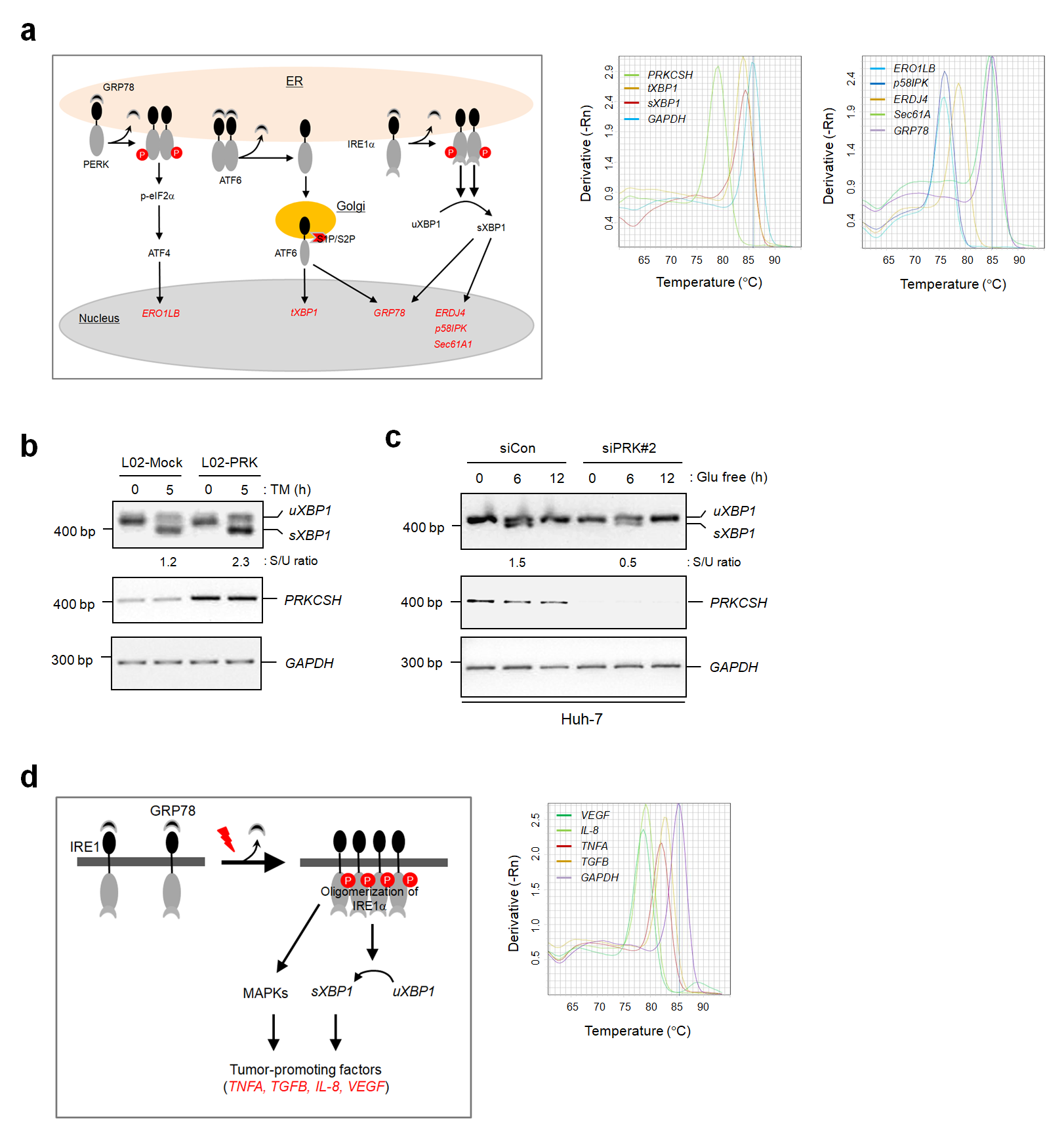


**Supplementary Figure 3** (related to Figs. 2, 3, and 8). Expression analysis of UPR-related genes and tumor-promoting factors. **a** Schematic illustration of three branches of UPR signaling and their target genes. The right part shows melting curves generated for each of the target genes to check for the presence of a non-specific PCR product or primer dimers, which would be visible as additional peaks. **b, c** Semi-quantitative RT-PCR analysis of *XBP1* mRNA splicing in (b) L02-Mock and L02-PRK cells and (c) PRKCSH*-*silenced Huh-7 cells treated with 10 μg/mL TM for the indicated time. *XBP1* mRNA splicing was estimated as the ratio of spliced *XBP1* mRNA (*sXBP1*) to unspliced *XBP1* mRNA (*uXBP1*) and the data normalized to the *GAPDH* gene are shown below the blots. **d** Schematic illustration of UPR-regulated tumor-promoting factors. The right part shows melting curves generated for each of the target genes to check for the presence of a non-specific PCR product or primer dimers.


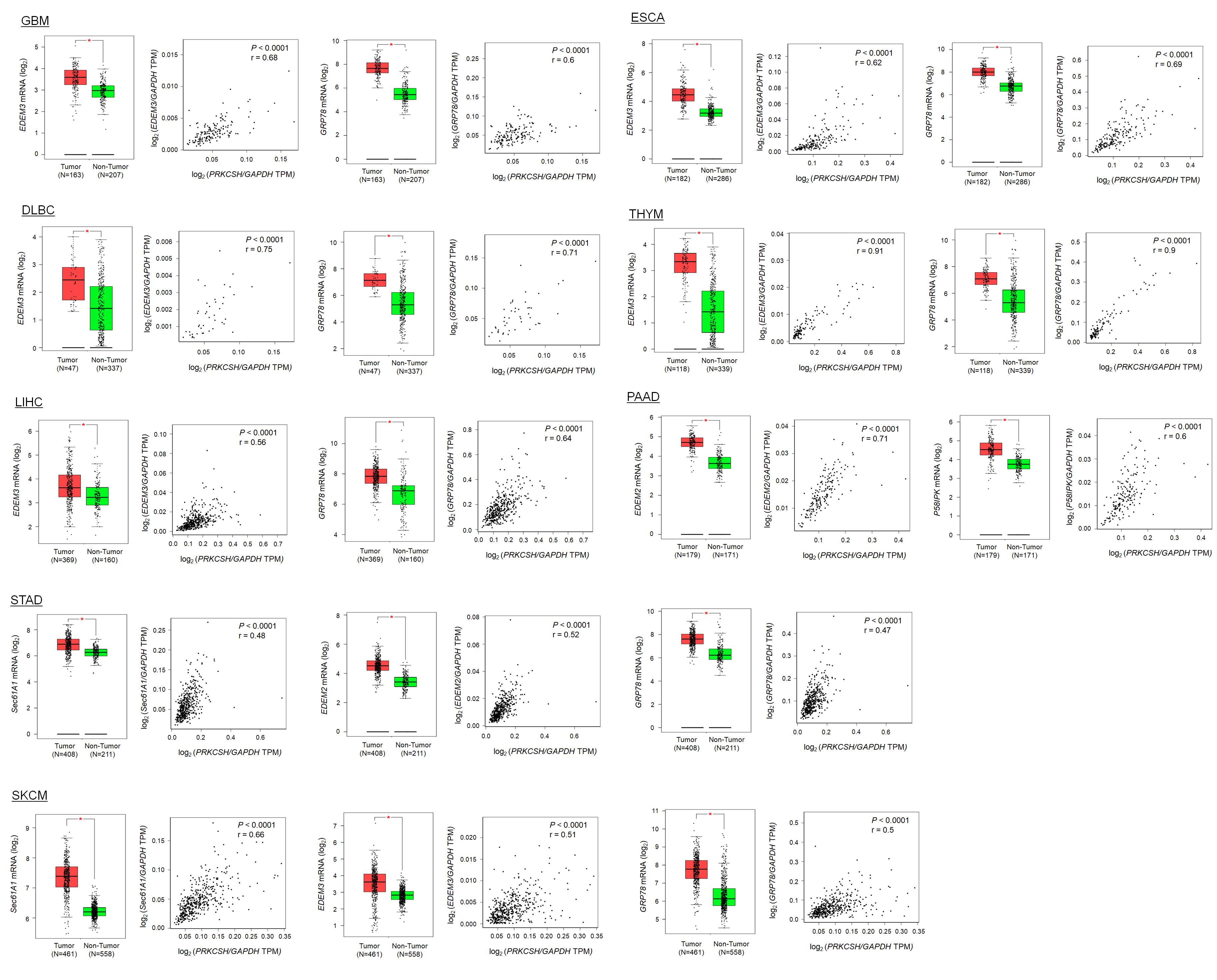


**Supplementary Figure 4** (related to Fig. 3). Expression level of IRE1α−XBP1 target genes in human tumor and non-tumor tissues and their correlation with the expression of *PRKCSH* mRNA in the same tissues. Gene expression data from TCGA were analyzed by using the GEPIA web tool. Scatter plots show relative expression of XBP1 target genes in non-tumor and tumor tissues. The median expression levels in each group are indicated by horizontal lines. Significance of the differences between non-tumor and tumor (left) was determined by One-way ANOVA (*P* < 0.001). Correlation between XBP1 target genes and PRKCSH (right) was determined by Pearson’s rank correlation coefficient. r Pearson correlation coefficient, *P* Pearson p-value.


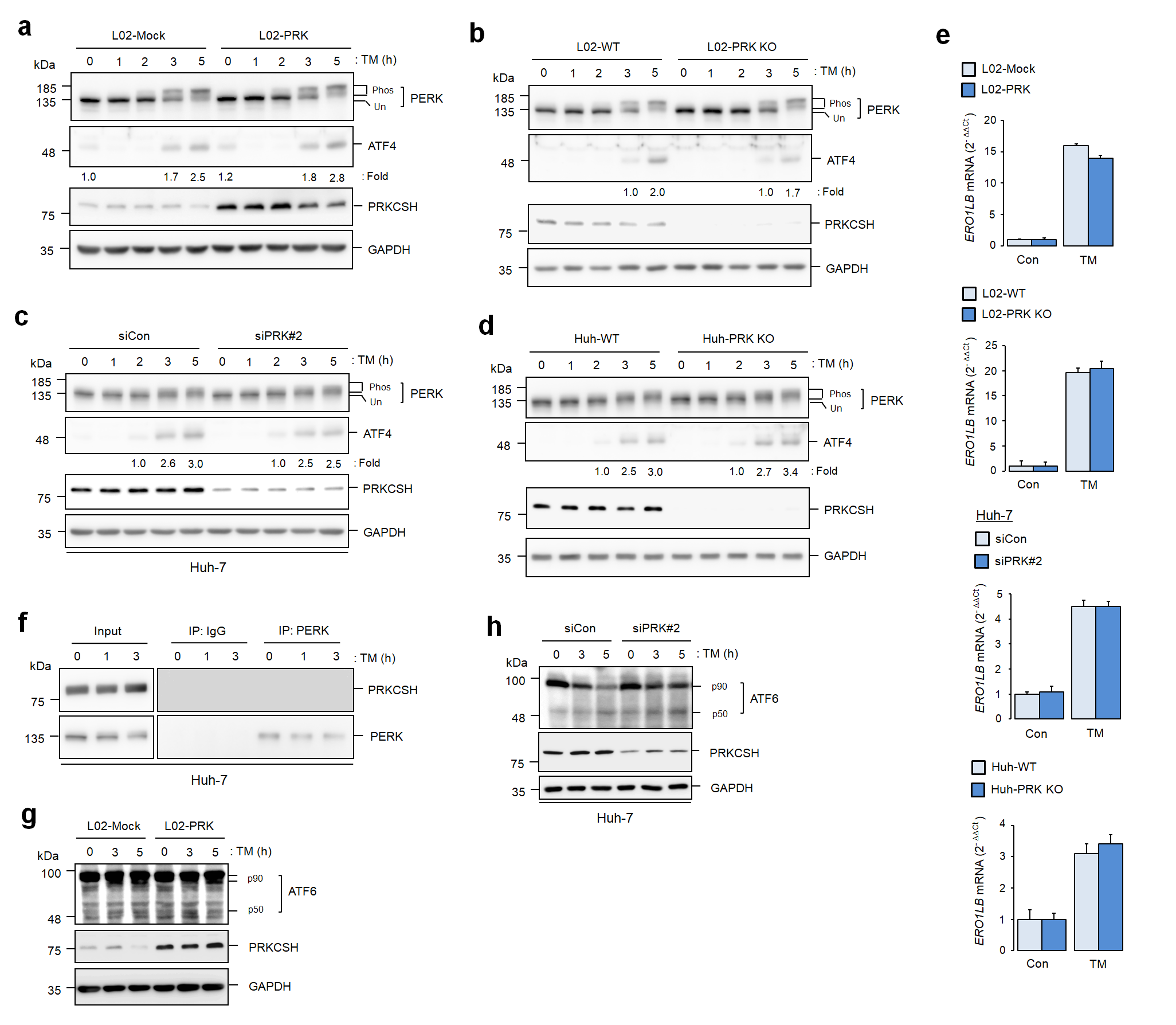


**Supplementary Figure 5** (related to Fig. 3 or Fig. 5). PRKCSH is not involved in the activation of PERK or ATF6 under ER stress condition. **a−d** Immunoblot analysis of PERK phosphorylation and ATF4 expression in L02-Mock and L02-PRK cells (a), L02-WT and L02-PRK KO cells (b), PRKCSH*-*silenced Huh-7 cells (c), and Huh-WT and Huh-PRK KO cells (d) treated with 10 μg/mL TM for the indicated time. GAPDH was used as a loading control. **e** Quantitative real-time PCR analysis of *ERO1LB* mRNA (a PERK/ATF4 -target gene) in L02-Mock and L02-PRK cells, L02-WT and L02-PRK KO cells, PRKCSH-silenced Huh-7 cells and Huh-WT and Huh-PRK KO cells after treatment with 10 μg/mL TM for 5 h. Data are shown as mean ± SEM of 4 independent experiments. *P* values were calculated by ANOVA. ***P* < 0.01. **f** Immunoblot analysis of complex formation between endogenous PRKCSH and PERK. Immunoprecipitates were prepared from L02 cells treated with 10 μg/mL TM for the indicated time. Immunoprecipitation was performed by using anti-PERK antibody and normal rabbit IgG as control antibody. **g** Immunoblot analysis of ATF6 activation in L02-Mock and L02-PRK cells treated with 10 μg/mL TM for the indicated time. **h** Immunoblot analysis of ATF6 activation in PRKCSH-silenced Huh-7 cells treated with 10 μg/mL TM for the indicated time.


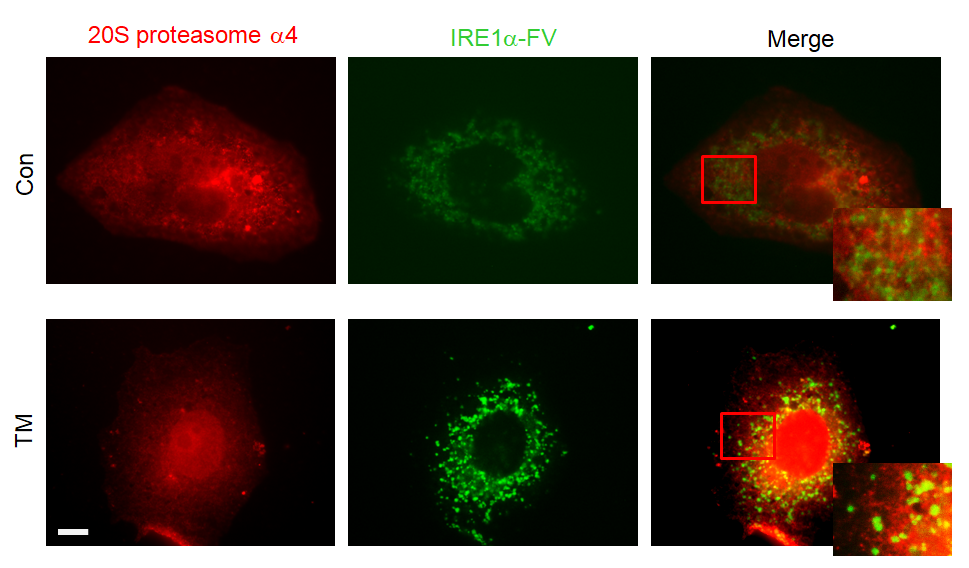


**Supplementary Figure 6** (related to Fig. 4). Assay for co-localization of oligomerized IRE1α with the proteasome. Immunocytochemical analysis of Huh-7 cells ectopically expressing IRE1α-FV and treated with or without 10 μg/mL TM for 5 h. Cells were immunostained with anti-20S proteasome α4 antibody. Oligomerized IRE1α was not associated with the proteasome. Scale bar represents 5 µm.


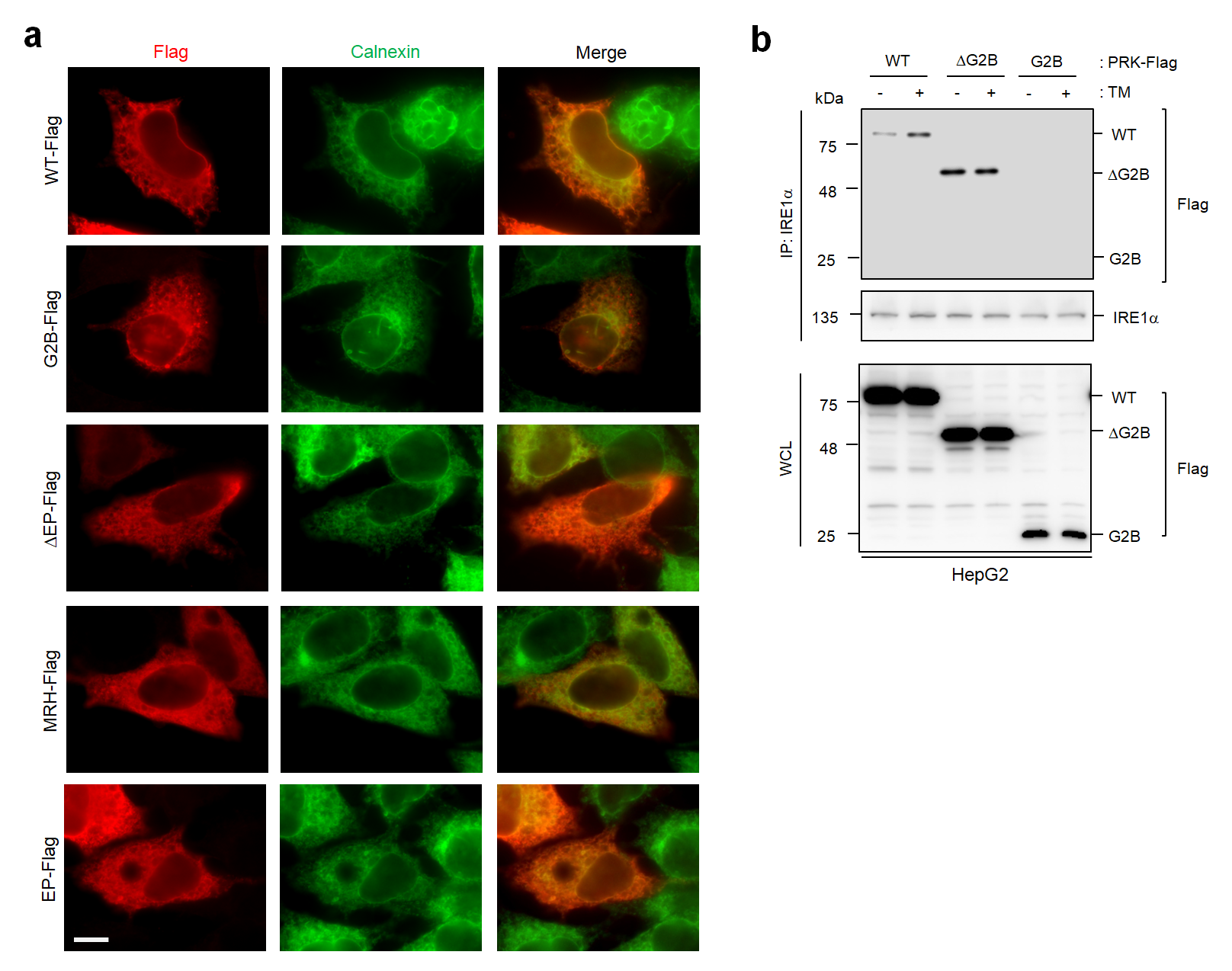


**Supplementary Figure 7** (related to Fig. 5). Intracellular localization of WT and mutant PRKCSHs and analysis of complex formation between the G2B mutant and IRE1α. **a** Immunocytochemical analysis of Flag-tagged WT and mutant PRKCSH in transfected HepG2 cells. Calnexin was used as an ER marker. Scale bar represents 5 µm. **b** Immunoblot analysis of complex formation between endogenous IRE1α and Flag-tagged mutant PRKCSHs. Immunoprecipitates were prepared from transfected HepG2 cells treated with 10 μg/mL TM for 1 h.


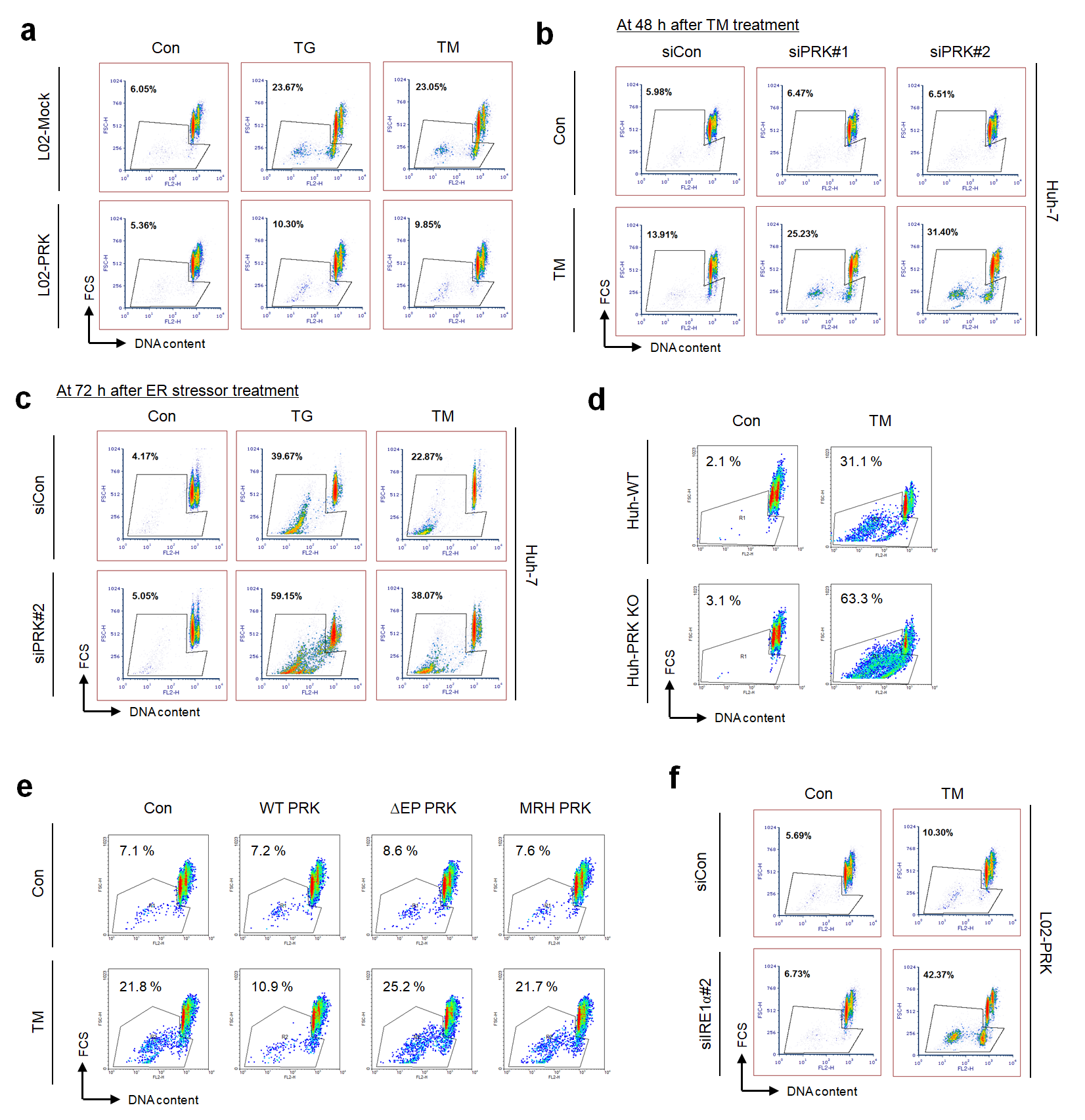


**Supplementary Figure 8** (related to Fig. 7). Analysis of ER stress–mediated apoptosis. **a** Mock or PRK cells treated with 10 μg/mL TM or 1 μM thapsigargin (TG) for 72 h. Cell death was determined using PI staining followed by FACS analysis. **b** PRKCSH-silenced Huh-7 cells treated with 10 μg/mL TM for 48 h. **c** PRKCSH-silenced Huh-7 cells treated with 10 μg/mL TM or 1 μM TG for 72 h. **d** Huh-WT and Huh-PRK KO cells treated with 10 μg/mL TM for 72 h. **e** L02 cells transfected with the control vector, WT PRKCSH, ΔE/P mutant, or MRH mutant; cells were treated with 10 μg/mL TM for 72 h. **f** IRE1α-silenced L02-PRK cells treated with 10 μg/mL TM for 72 h. Apoptotic cells are characterized by two populations: that with chromatin fragmentation and that with nuclear condensation.


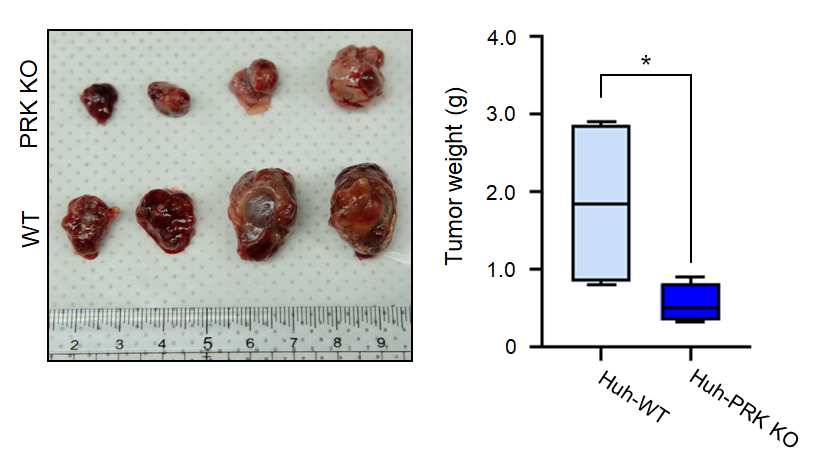


**Supplementary Figure 9** (related to Fig. 7). Analysis of *in vivo* tumor growth in Huh-PRK KO or WT cell-bearing nude mice. Picture shows tumor sizes of each group (left), and graph shows the tumor weights (right). The center line of the boxplots denotes the median, the bounds of the box indicate 25 to 75% and the whiskers represent 5 to 95 %, respectively. Significance of the differences between the two categories was determined by the Student *t*-test (**P* < 0.05).


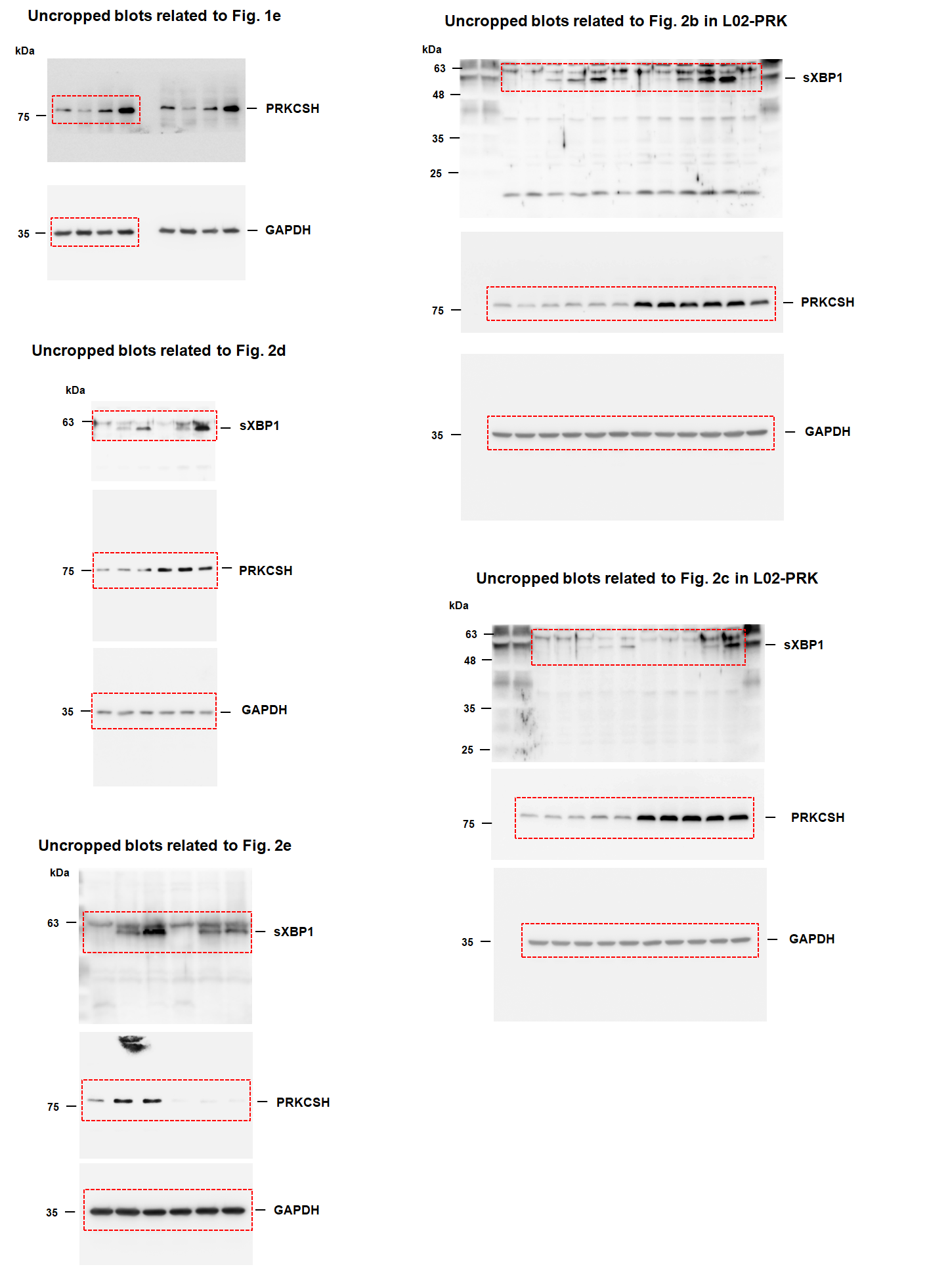


**Supplementary Figure 10.** Unprocessed data for immunoblots and agarose gels of PCR products.


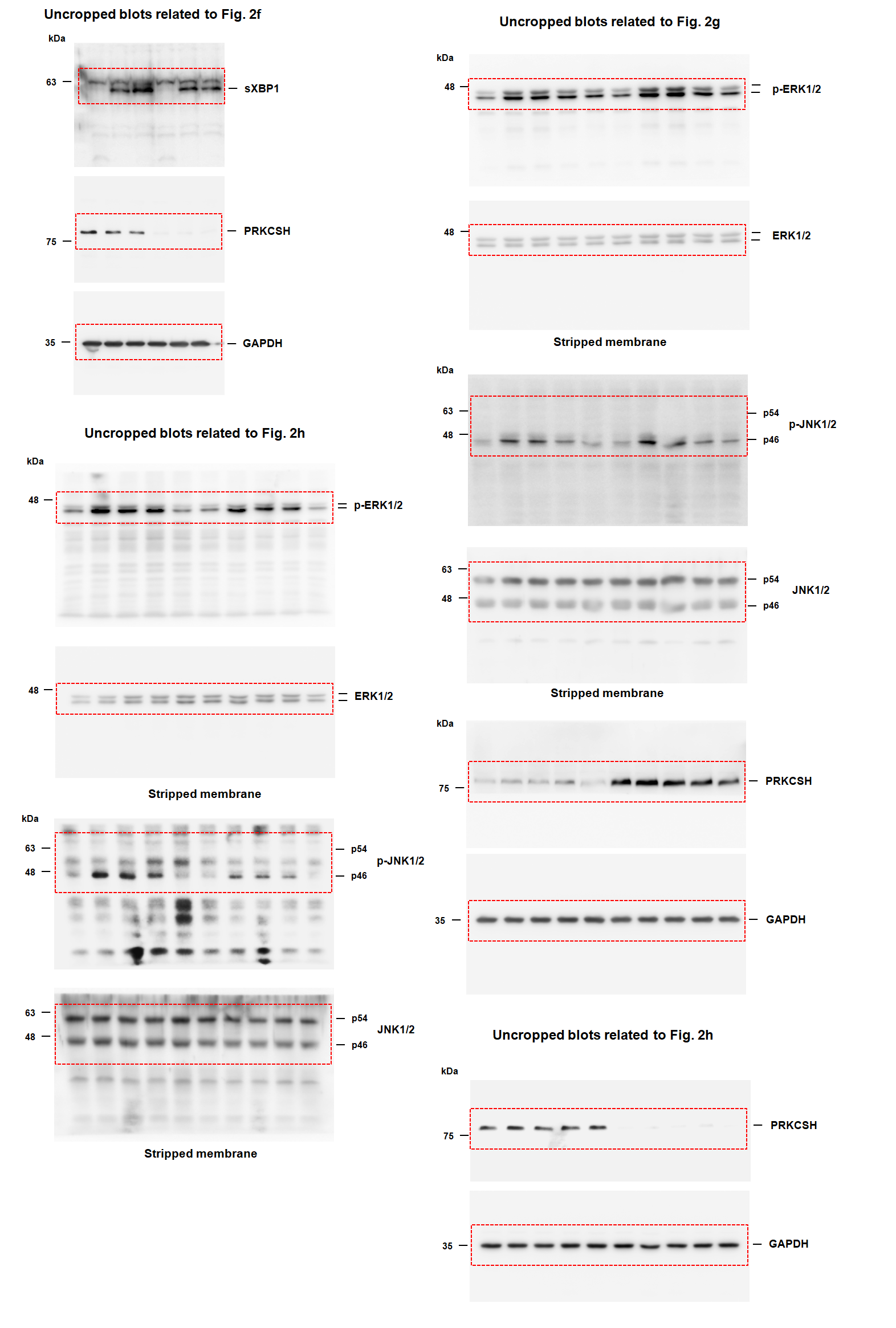


Supplementary Figure 10. Continued


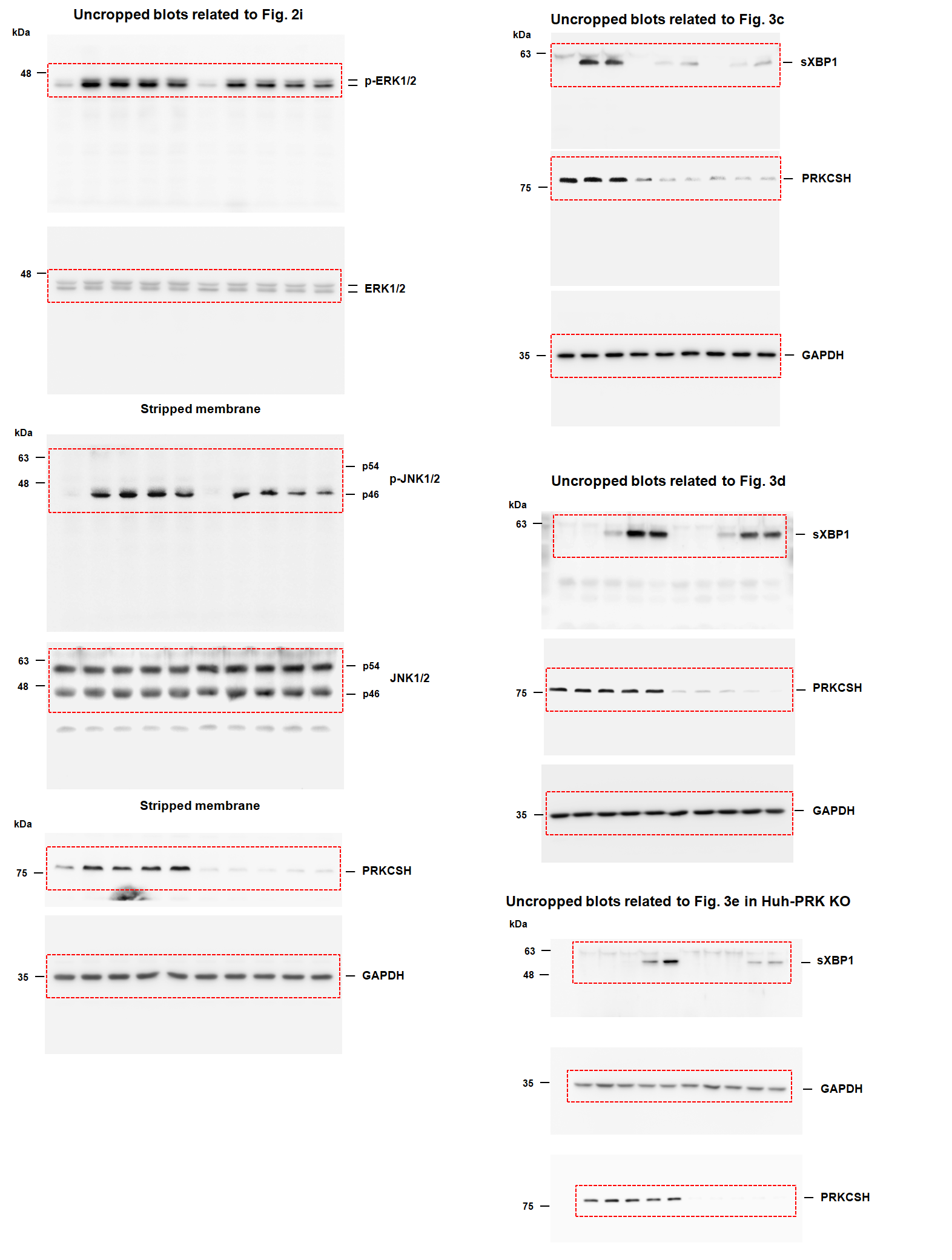


Supplementary Figure 10. Continued


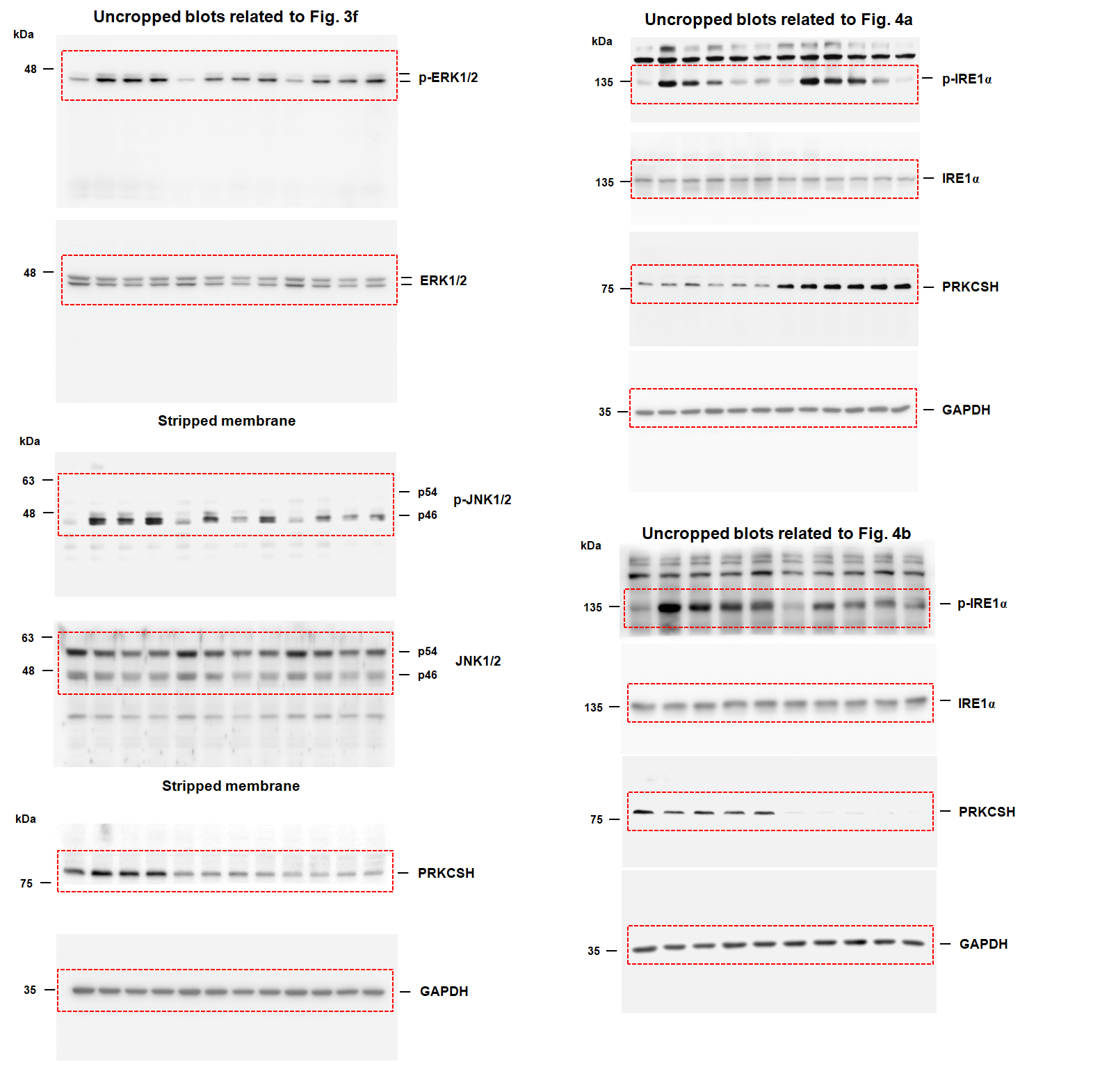


Supplementary Figure 10. Continued


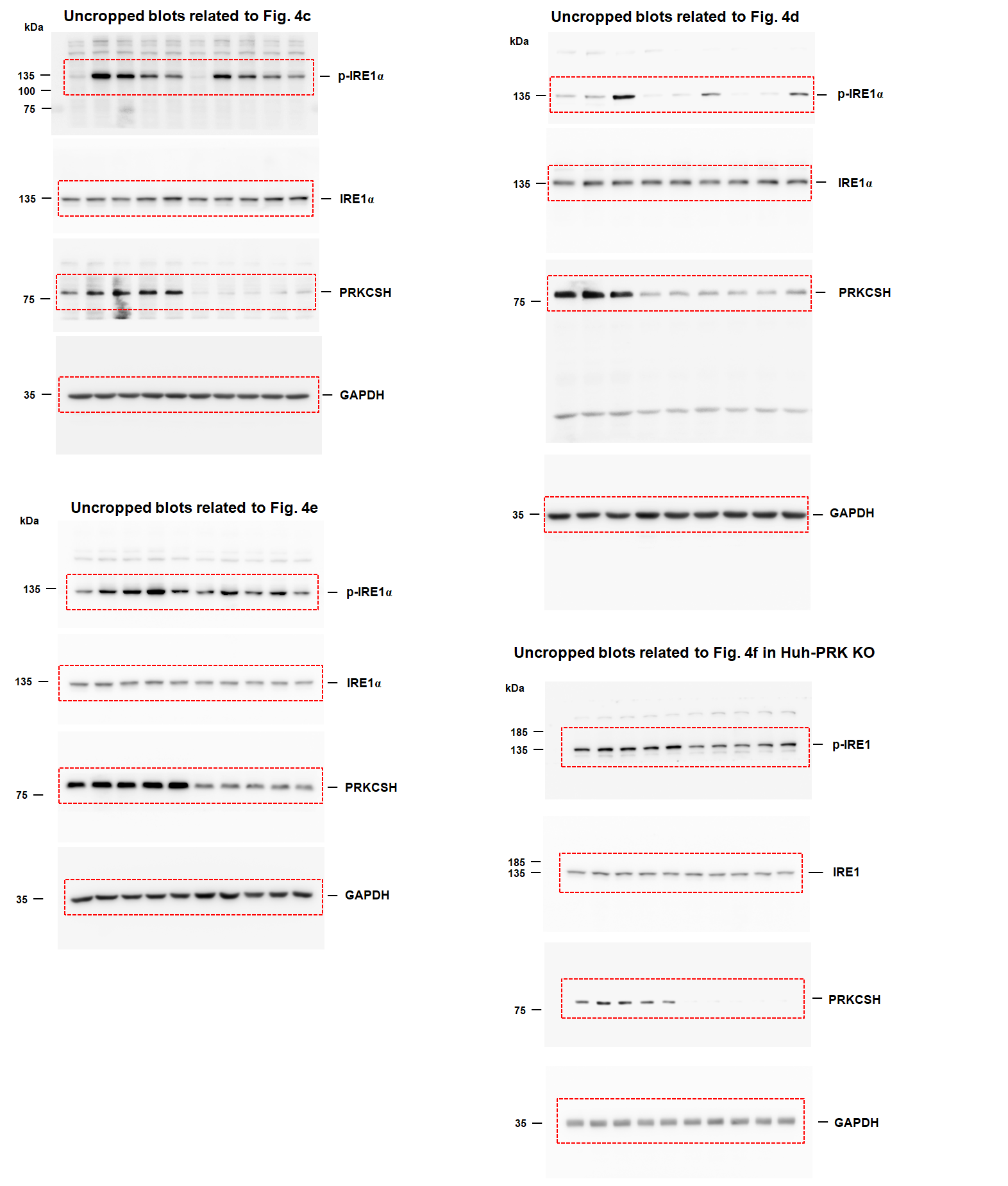


Supplementary Figure 10. Continued


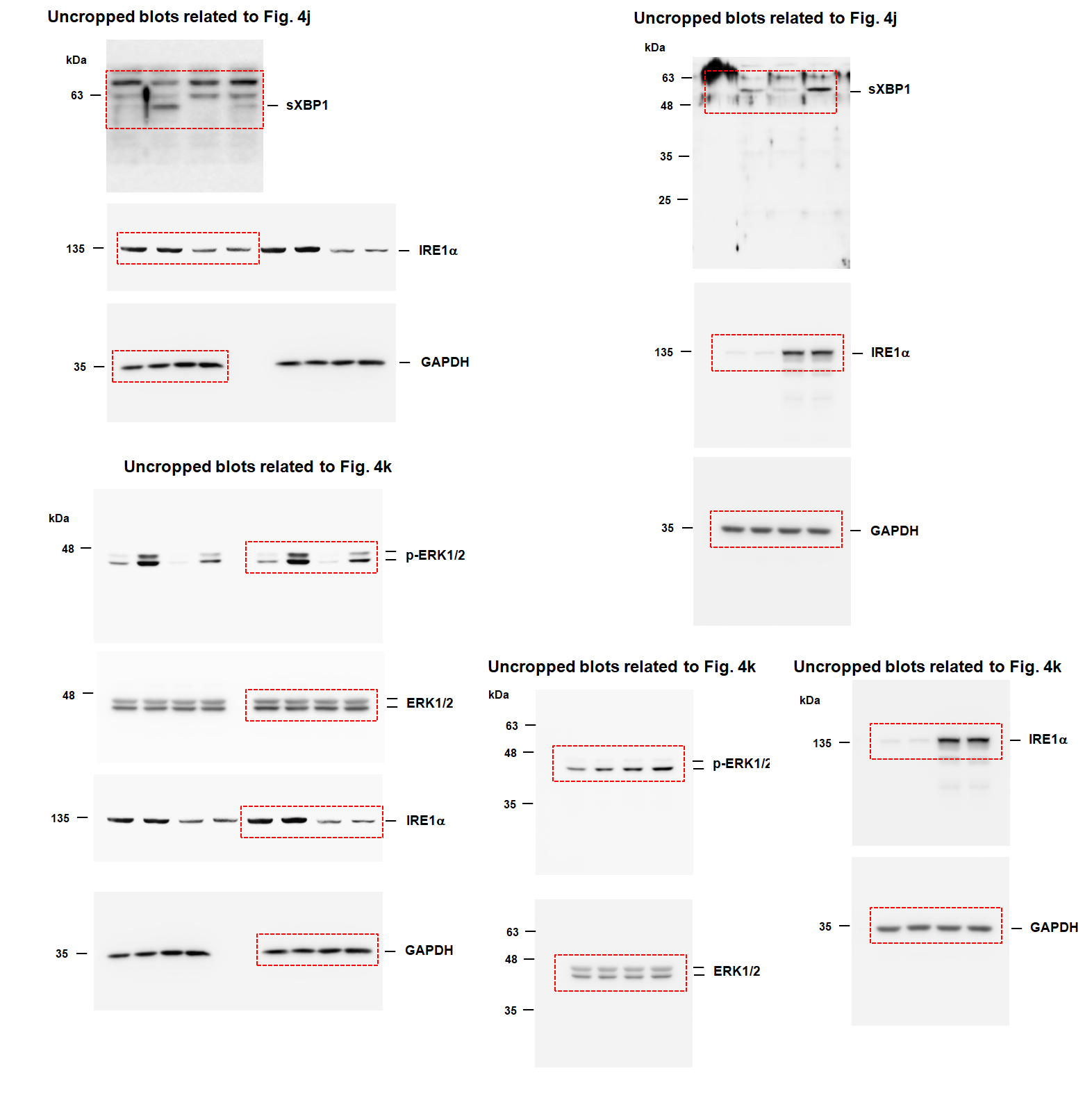


Supplementary Figure 10. Continued


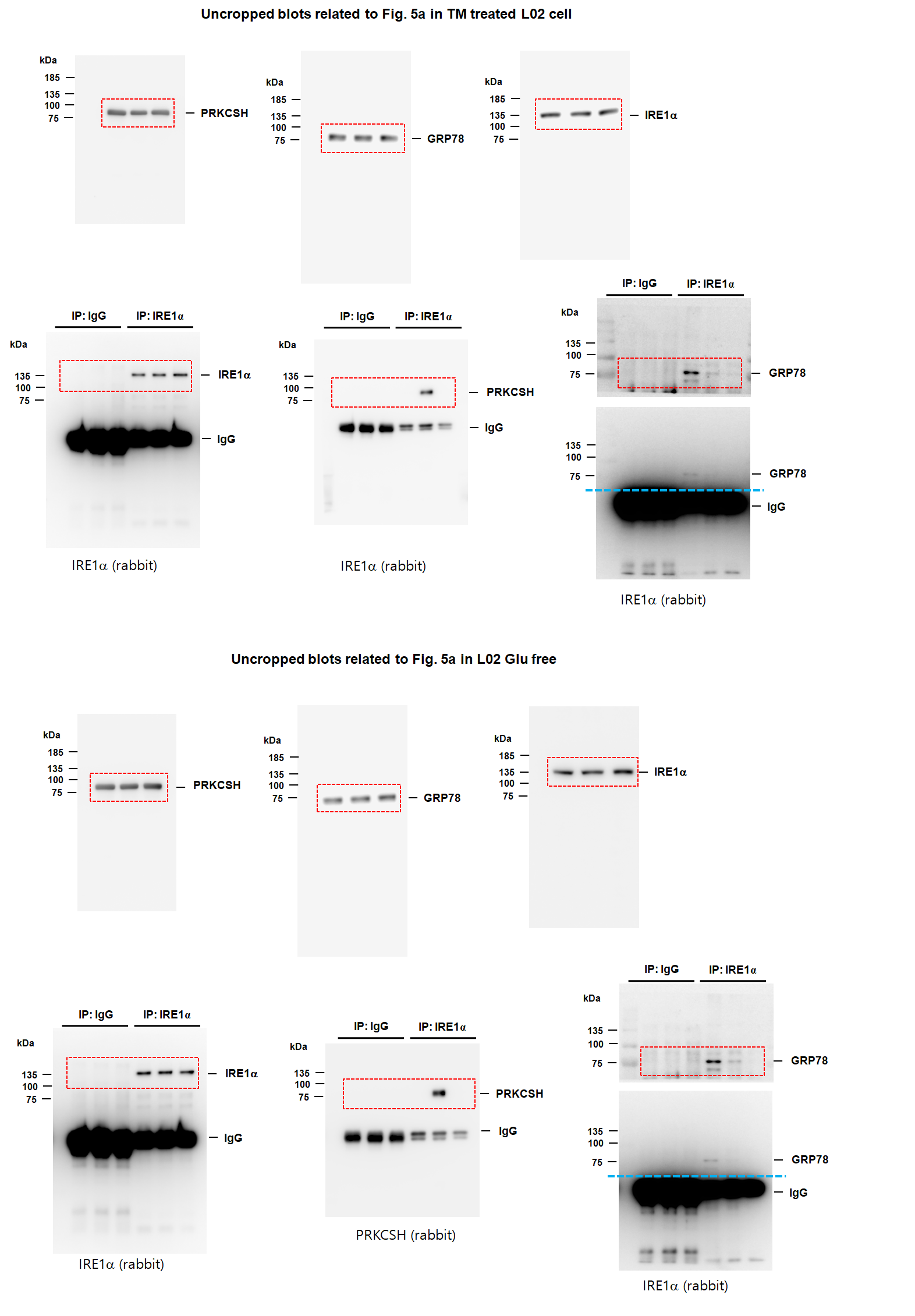


Supplementary Figure 10. Continued


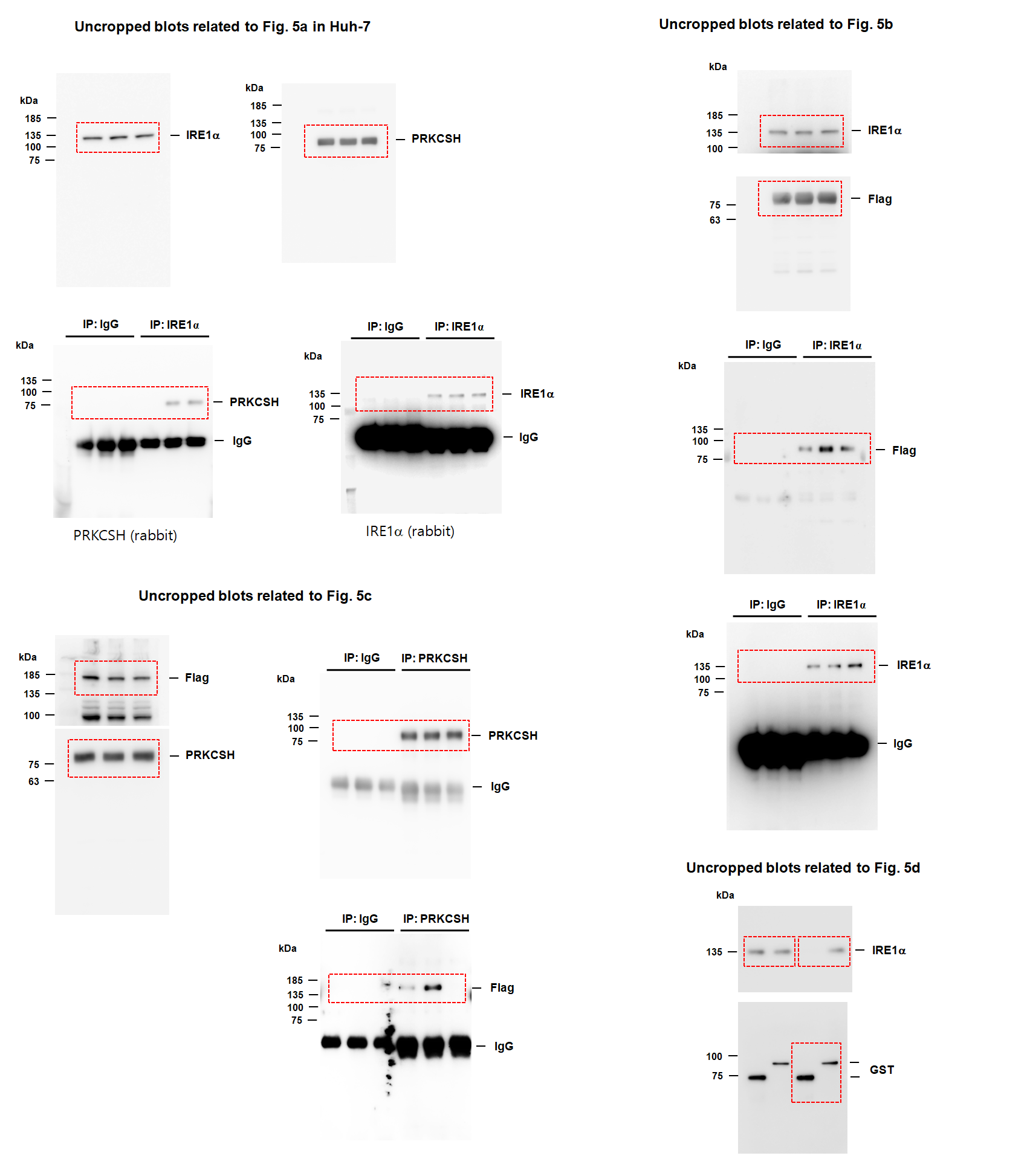


Supplementary Figure 10. Continued


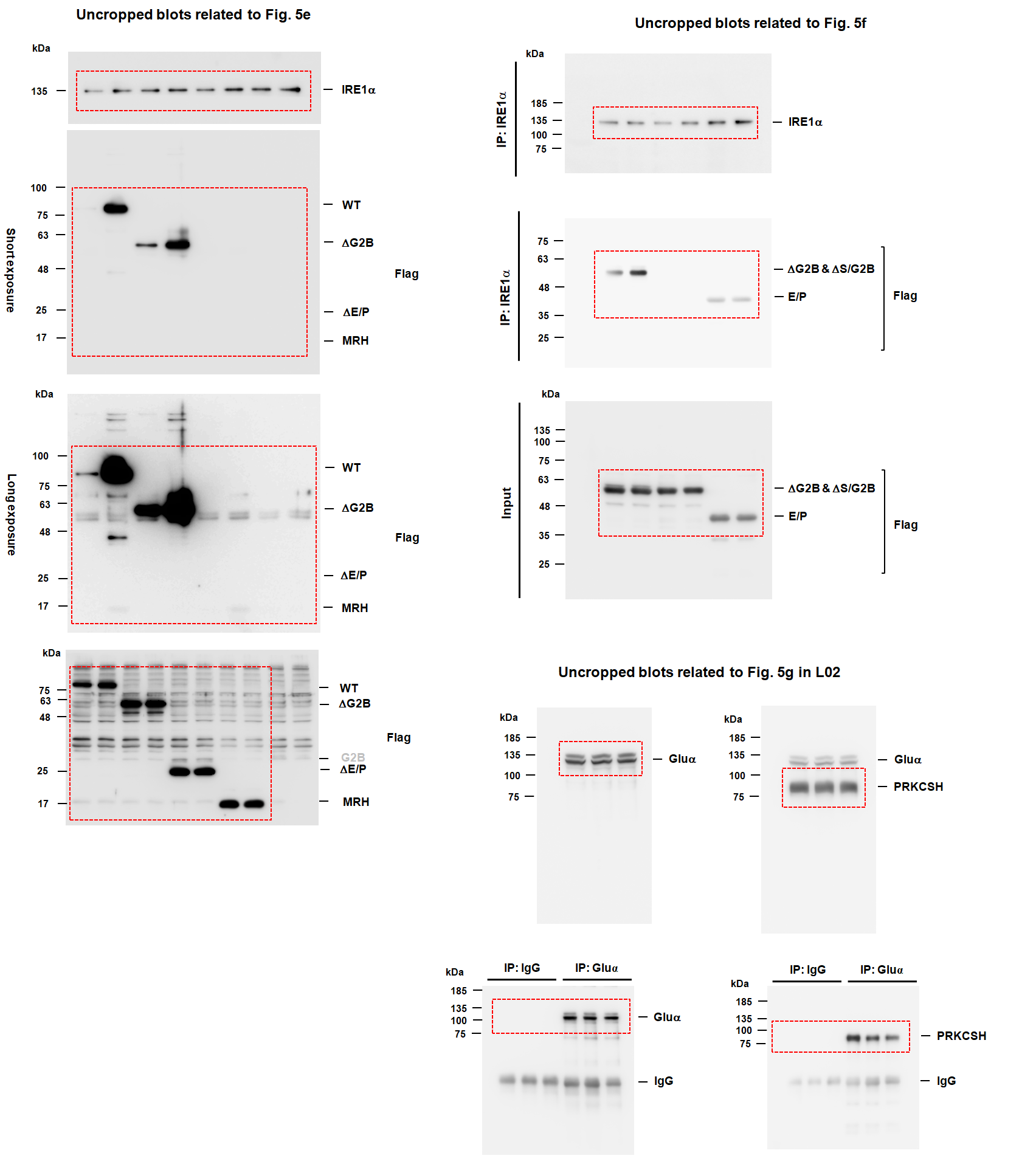


Supplementary Figure 10. Continued


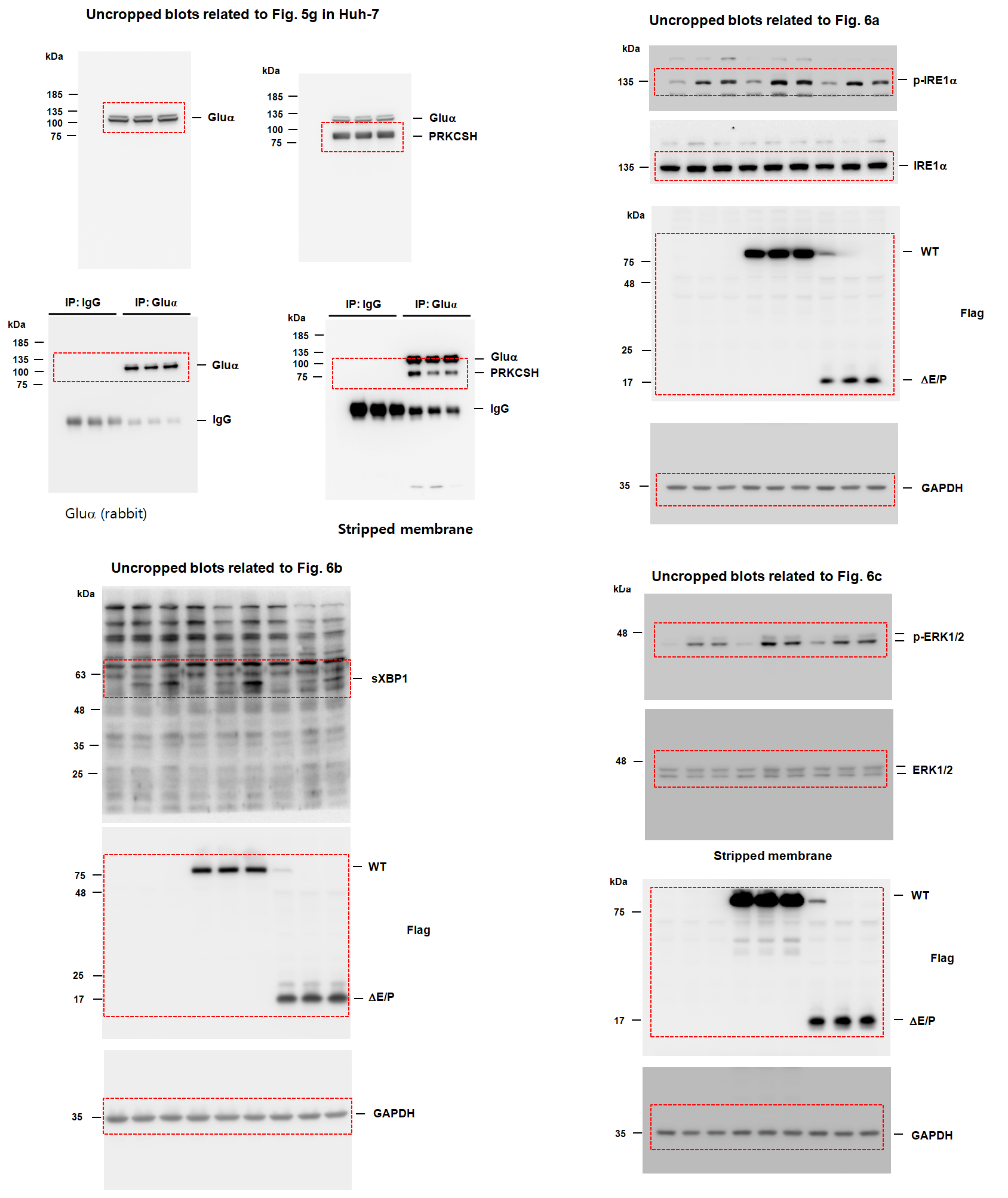


Supplementary Figure 10. Continued


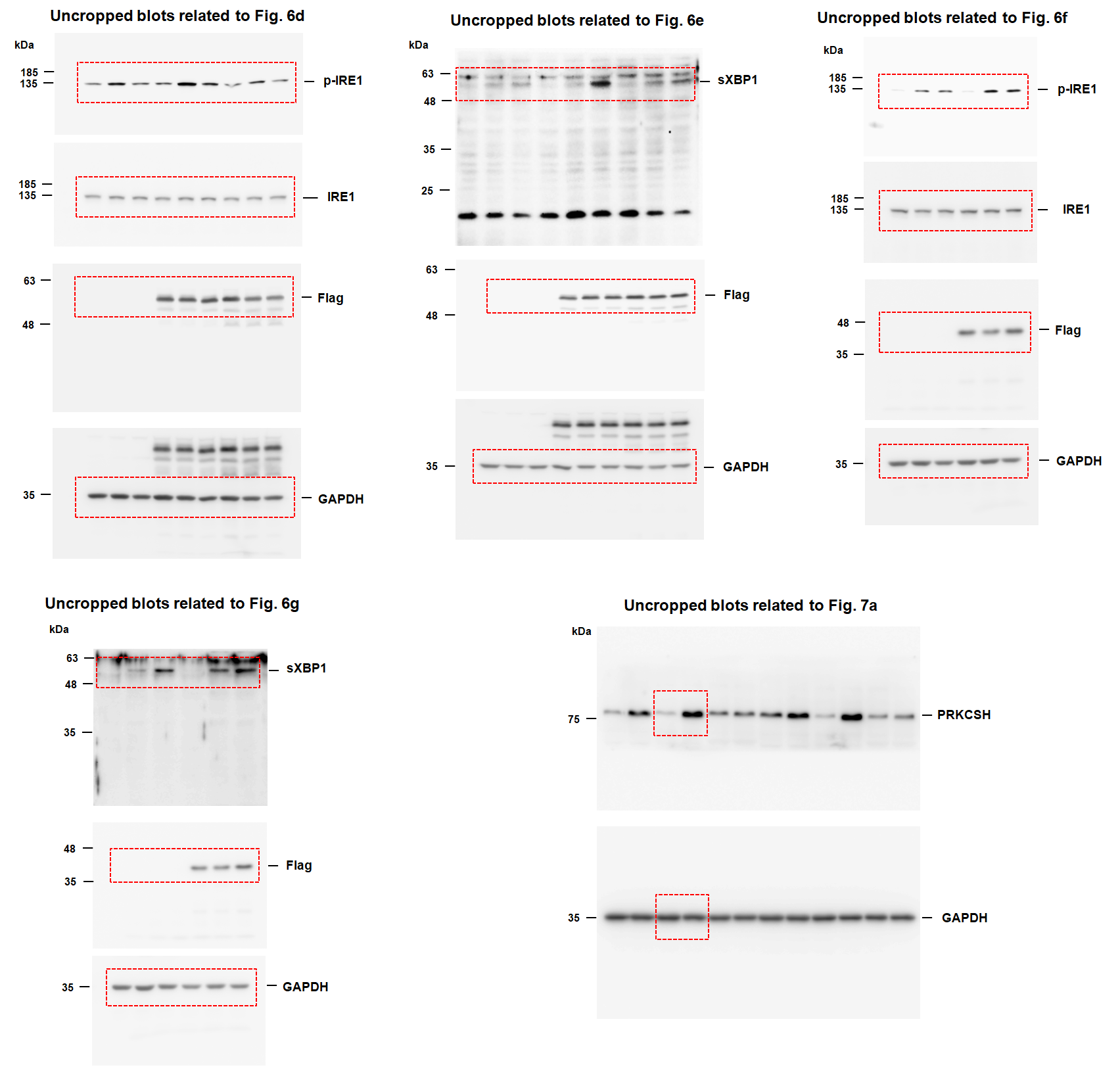


Supplementary Figure 10. Continued


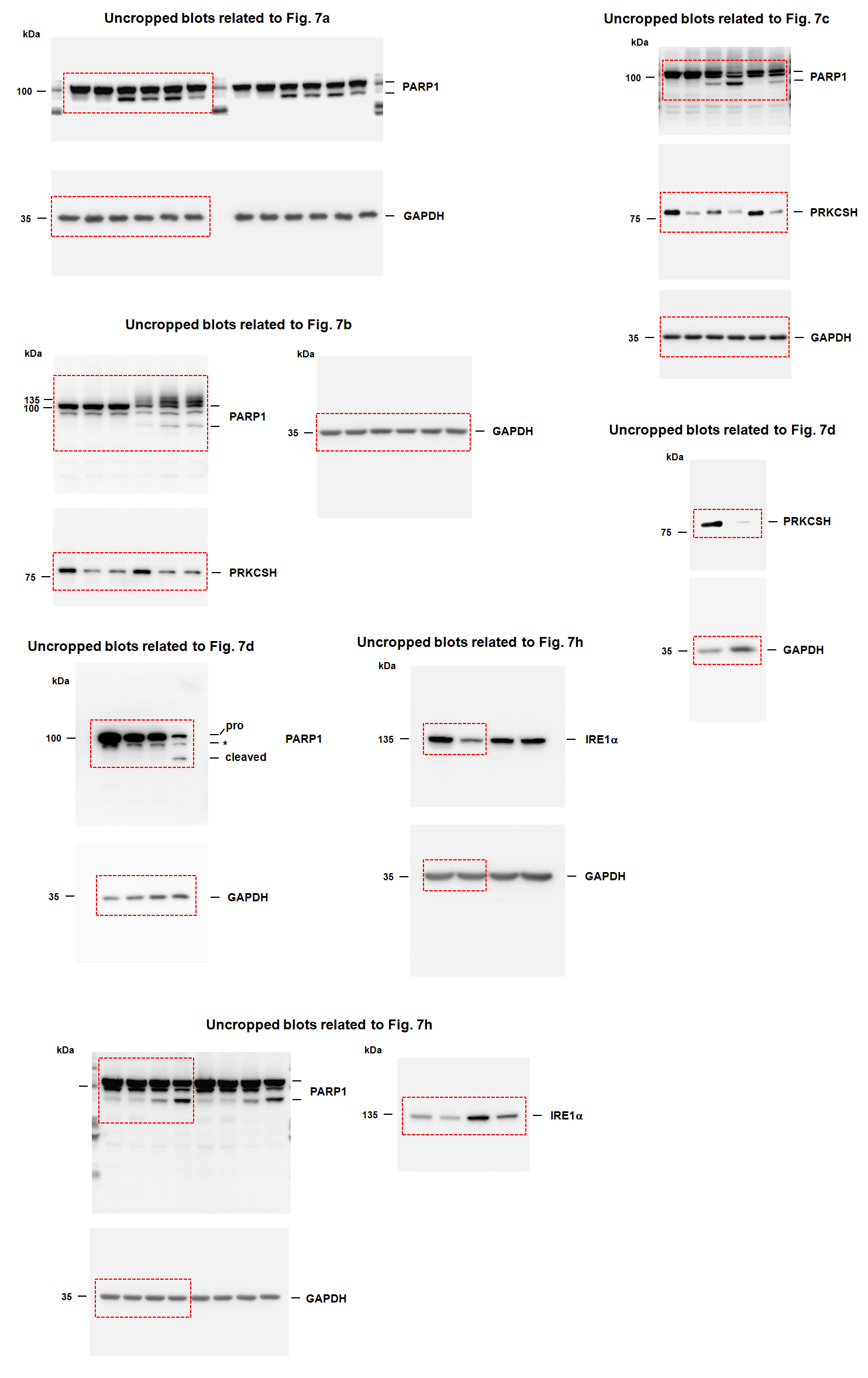


Supplementary Figure 10. Continued


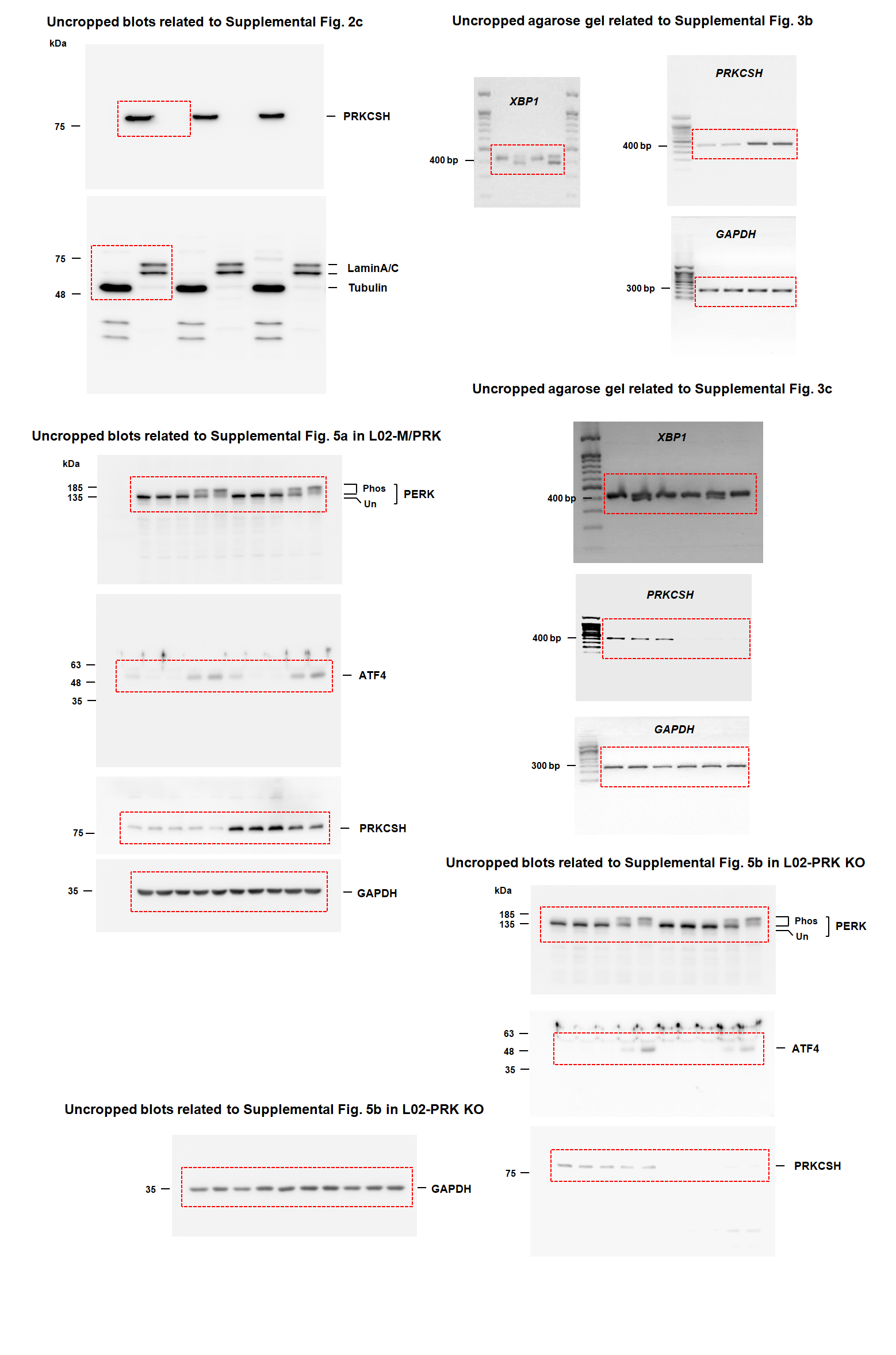


Supplementary Figure 10. Continued


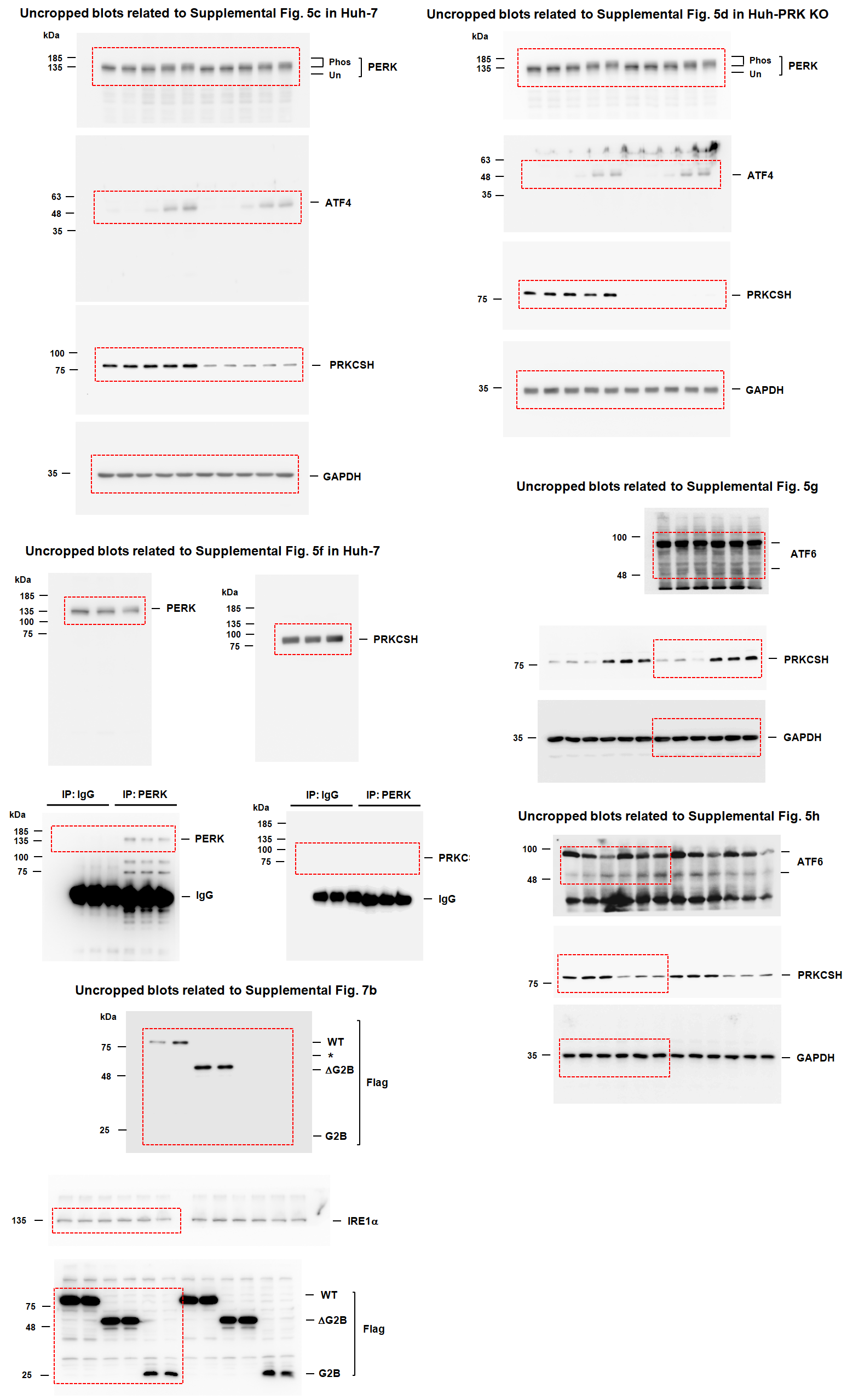


Supplementary Figure 10. Continued

**Supplementary Tables**

Supplementary Table 1. Relationship between PRKCSH expression in HCC tissues and clinicopathological features of 58 patients (related to main Fig. 1)

| Feature | Number of patients | PRKCSH staining | | | *P* value |
| --- | --- | --- | --- | --- | --- |
|  |  | Positive, *n* (%) | Negative, *n* (%) | |  |
| Sex |  |  | |  | 0.412 |
| Male | 43 | 36 (80) | | 9 (69) |  |
| Female | 15 | 9 (20) | | 4 (31) |  |
| Age | 20−75, median = 53.25 | | | | 0.821 |
| ≤ 60 year | 42 | 33 (72) | | 9 (75) |  |
| > 60 year | 16 | 13 (28) | | 3 (25) |  |
| Tumor size (cm) |  |  | |  | 0.478 |
| < 5 | 25 | 17 (50) | | 8 (62) |  |
| ≥ 5 | 21 | 17 (50) | | 4 (38) |  |
| Not available | 12 | 12 | | 0 |  |
| Differentiation |  |  | |  | 0.930 |
| Well | 7 | 5 (21) | | 2 (22) |  |
| Moderate to poor | 26 | 19 (79) | | 7 (78) |  |
| Not available | 25 | 22 | | 3 |  |
| Invasion depth |  |  | |  | 0.478 |
| T1+T2 | 25 | 17 (50) | | 8 (62) |  |
| T3+T4 | 21 | 17 (50) | | 4 (38) |  |
| Not available | 12 | 12 | | 0 |  |
| Lymph nodes metastasis |  |  | |  | 0.512 |
| N0 | 42 | 30 (97) | | 12 (100) |  |
| N1 | 1 | 1 (3) | | 0 (0) |  |
| Not available | 15 | 15 | | 0 |  |
| **Extrahepatic metastasis** |  |  | |  | **0.029** |
| No | 45 | 33 (72) | | 12 (100) |  |
| Yes | 13 | 13 (28) | | 0 (0) |  |
| **TNM stage** |  |  | |  | **0.028** |
| I + II | 25 | 17 (36) | | 8 (72) |  |
| III + IV | 33 | 30 (63) | | 3 (27) |  |

Supplementary Table 2. List of primers used in qPCR

| Gene | Primer sequences | Reference |
| --- | --- | --- |
| PRKCSH | Forward 5’-GGCGTCTCCCTCACCAATCATC-3’ | This study |
|  | Reverse 5’-TCTCCTCCCGTGCCTTCTTCCAGT-3’ |  |
| XBP1 | Forward 5’-CTGGAACAGCAAGTGGTAGA-3’ | 1 |
|  | Reverse 5’-CTGGGTCCTTCTGGGTAGAC-3’ |  |
| sXBP1 | Forward 5’-CGCTTGGGGATGGATGCCCTG-3’ | 2 |
|  | Reverse 5’-CCTGCACCTGCTGCGGACT-3’ |  |
| tXBP1 | Forward 5’-GGCATCCTGGCTTGCCTCCA-3’ | 2 |
|  | Reverse 5’-GCCCCCTCAGCAGGTGTTCC-3’ |  |
| GAPDH | Forward 5’-ATCATCCCTGCCTCTACTGG-3’ | This study |
|  | Reverse 5’-TGGGTGTCGCTGTTGAAGTC-3’ |  |
| GRP78 | Forward 5’-TGACATTGAAGACTTCAAAGCT-3’ | 2 |
|  | Reverse 5’-CTGCTGTATCCTCTTCACCAGT-3’ |  |
| ERDJ4 | Forward 5’-TCGGCATCAGAGCGCCAAATCA-3’ | 2 |
|  | Reverse 5’-ACCACTAGTAAAAGCACTGTGTCCAAG-3’ |  |
| ERO1LB | Forward 5’-TTCTGGATGATTGCTTGTGTGAT-3’ | 2 |
|  | Reverse 5’-GGTCGCTTCAGATTAACCTTGT-3’ |  |
| Sec61A1 | Forward 5’-TGTCATCTCCCAAATGCTCTCA-3’ | 3 |
|  | Reverse 5’-ACAGGTAATAGCAAAGGCCAC-3’ |  |
| P58IPK | Forward 5’-TGTGTTTGGGATGCAGAACTAC-3’ | 3 |
|  | Reverse 5’-TCTTCAACTTTGACGCAGCTT-3’ |  |
| TNFA | Forward 5’-GAGGCCAAGCCCTGGTATG-3’ | This study |
|  | Reverse 5’-CGGGCCGATTGATCTCAGC-3’ |  |
| IL-8 | Forward 5’-ACTGAGAGTGATTGAGAGTGGAC-3’ | This study |
|  | Reverse 5’-AACCCTCTGCACCCAGTTTTC-3’ |  |
| VEGF | Forward 5’-AGGGCAGAATCATCACGAAGT-3’ | This study |
|  | Reverse 5’-AGGGTCTCGATTGGATGGCA-3’ |  |

Supplementary Table 2. continued

| Gene | Primer sequences | References |
| --- | --- | --- |
| PRKCSH-Flag | Forward 5’- CGCGGATCCATGCTGTTGCCGCTGCTGCT-3’ | This study |
|  | Reverse 5’-TGCTCTAGACTACTTATCGTCGTCATCCTTGTAATC  GAGCTCGTCATGGTCGTCTT-3’ |  |
| Non-tag PRKCSH | Forward 5’-CGCGGATCCATGCTGTTGCCGCTGCTG-3’ | This study |
|  | Reverse 5’- TGCTCTAGACTAGAGCTCGTCATGGTCGTCTT-3’ |  |
| PRK delG2B | Forward 5’- CGCGGATCCATGCTGTTGCCGCTGCTGCTGCTGC  TACCCATGTGCTGGGCCGTGAAGCCAGAGAGAGAGGCCAAAGAG-3’ | This study |
|  | Reverse 5’- TGCTCTAGACTGCTTATCGTCGTCATCCTTGTAAT  CGAGCTCGTCATGGTCGTCTTCG-3’ |  |
| PRK delS/G2B | Forward 5’- CGCGGATCC TGAAGCCAGAGAGAGAGGCCAAA  GAG-3’ | This study |
| PRK delEP | Forward 5’- CGCGGATCCATGCTGTTGCCGCTGCTGCTGCTGC  TACCCATGTGCTGGGCCGTGCAGCCCAAGCCGGCCA-3’ | This study |
| PRK MRH | Forward 5’- CGCGGATCCATGCTGTTGCCGCTGCTGCTGCTGC  TACCCATGTGCTGGGCCGTGGAATACGTCTACCGCCTCTGCCC-3’ | This study |
| PRK G2B | Forward 5’- CGCGGATCCATGCTGTTGCCGCTGCTGC-3’ | This study |
|  | Reverse 5’- TGCTCTAGACTACTTATCGTCGTCATCCTTGTAAT  CGAGCTCGTCATGCTCAGCTTCCTCCTTCACTGTCCG-3’ |  |
| PRK EP | Forward 5’- CGCGGATCCATGCTGTTGCCGCTGCTGCTGCT  GCTACCCATGTGCTGGGCCGTGAAGCCAGAGAGAGAGGCCAAAGAG -3’ | This study |
|  | Reverse 5’- TGCTCTAGACTACTTATCGTCGTCATCCTTGTA  ATCGAGCTCGTCATGGTTGGTGGTGAGCTCGTAGCACTG-3’ |  |

**Supplementary References**

1. Li, J. *et al*. The unfolded protein response regulator GRP78/Bip is required for endoplasmic reticulum integrity and stress-induced autophagy in mammalian cells. *Cell. Death. Differ.* **15**, 1460-1471 (2008).
2. Van Galen, P. *et al*. The unfolded protein response governs integrity of the haematopoietic stem-cell pool during stress. *Nature*, **510**, 268-272 (2014).
3. Mimura, N. *et al*. Blockade of XBP1 splicing by inhibition of IRE1α is a promising therapeutic option in multiple myeloma. *Blood*. **119**, 5772-5781 (2012).
